# Pathogenic load and frailty in older adults: Singapore longitudinal ageing study

**DOI:** 10.18632/aging.104076

**Published:** 2020-11-06

**Authors:** Tze Pin Ng, Yanxia Lu, Crystal Tze Ying Tan, Qi Gao, Xinyi Gwee, Tamas Fulop, Anis Larbi

**Affiliations:** 1 Gerontology Research Programme, Department of Psychological Medicine, Yong Loo Lin School of Medicine, National University of Singapore, Singapore 119228, Singapore; 2 Singapore Immunology Network (SIgN), Agency for Science, Technology and Research (A*STAR), Singapore 138648, Singapore; 3 Department of Geriatrics, Faculty of Medicine, University of Sherbrooke, Sherbrooke QC J1K 2R1, Canada

**Keywords:** virus, immunosenescence, frailty, bacteria, mortality

## Abstract

Human evidence for the role of continuous antigenic stimulation from persistent latent infections in frailty is limited. We conducted a nested case-control study (99 deceased and 43 survivors) of participants aged 55 and above in a longitudinal ageing cohort followed up from 2003 to 2017. Using blood samples and baseline data collected in 2003-2004, we examined the association of pathogenic load (PL) count of seropositivity to 10 microbes (viruses, bacteria and mycoplasma) with cumulated deficit-frailty index (CD-FI) and the physical frailty (PF) phenotype, and mortality. Controlling for age, sex, education, smoking and alcohol histories, high PL (7-9) versus low PL (3-6) was associated with an estimated increase of 0.035 points in the CD-FI (Cohen’s D=0.035 / 0.086, or 0.41). High PL was associated with 8.5 times odds of being physically frail (p=0.001), 2.8 times odds of being weak (p=0.010), 3.4 times odds of being slow (p=0.024), and mortality hazard ratio of 1.53 (p=0.046). There were no significant associations for specific pathogens, except marginal associations for Epstein-Barr virus and Chikungunya. Conclusion: A high pathogenic load of latent infections was associated with increased risks of frailty and mortality.

## INTRODUCTION

Frailty is an age-associated state of health characterized by increased vulnerability to the effects of stress resulting eventually in increased risks of adverse health outcomes including multi-morbidity, cognitive impairment, disability, hospitalization, institutionalization and mortality [1, 2]. Frailty may be viewed as a pathophysiological state of diminished ability to maintain homeostasis due to increased allostatic load from chronic stress responses [3–6]. Physiological dysregulation in multiple organ systems underlying the concept of frailty [7–9] is the basis for two main approaches to measuring frailty in clinical research. One approach is the frailty index, an accumulated deficits construct using cumulated counts of age-related health deficits across multiple physiological and functional systems, and encompasses multiple dimensions of physical, psychological, biological and social functioning [10]. The other approach is the physical frailty phenotype, [11] clinically defined by the presence of unintentional weight loss, self-reported exhaustion, weakness, slowness, and low levels of physical activity. Frailty may therefore be examined globally or in terms of multiple phenotypes or domains.

The causes of age-related frailty are multifactorial, and chronic systemic inflammation is believed to play a central pathogenic role. The systemic pro-inflammatory milieu seen in old age is partly the result of the dysregulation of immune response associated with ageing (“immunosenescence”) involving both innate and (more extensively studied) adaptive immunity, suggesting the erosion of immune surveillance over time [12]. This is phenotypically characterized by the accumulated number and proportion of late-stage differentiated T cells, especially CD8+T cells. T cells may be driven to late differentiation and/or exhaustion by the continuous antigen stimulation from persistent latent infections from microbial agents. Persistent and latent infections which are highly prevalent worldwide include those caused by Herpesvirus such as herpes simplex viruses 1 and 2 (HSV-1 and HSV-2) and varicella-zoster virus (VZV); cytomegalovirus (CMV), Epstein-Barr virus (EBV), and other viruses such as hepatitis B virus (HBV), human papilloma virus (HPV), human immunodeficiency virus (HIV), as well as other microbes such as helicobacter pylori and mycoplasma pneumoniae.

Evidence for the role of continuous antigenic stimulation from persistent and latent infections in human frailty is limited. HIV patients show characteristic surface markers and functionality profiles on CD4+ or CD8+ T cells known to drive premature immunosenescence [13, 14]. High levels of CD8+ and low levels of CD4+ T-cells contributing to pro-inflammatory cytokine increase is reported to predict frailty in older adults [15] CMV infection is the most well studied viral factor driving the differentiation of the CD8+ T cells towards immunosenescence (Fulop et al, 2013), but human studies that examined the association between persistent CMV infection and clinical outcomes have produced contradictory results. Some studies have shown that CMV was associated with prevalent frailty, [16–18] functional and cognitive impairment, [19–21] and all-cause and cardiovascular disease mortality [22–24] but other studies have not supported these findings [15–28]. A recent systematic review and meta-analysis of six studies [16–18, 26, 27, 29] analysing the association between CMV, EBV, VZV, and HSV seropositivity and frailty in older people found an association between CMV infection and frailty only in older persons aged 60–79 years, but not in the oldest-old subjects. No association was found between EBV, VZV, and HSV infections and frailty [30].

So far, studies have selectively investigated less than a handful of individual viruses singly for their individual associations with frailty and related outcomes, and no studies have considered latent infections by bacterial or other pathogens. In this study, we assessed latent infection by ten viral, bacterial and mycoplasma agents (Cytomegalovirus, Herpes simplex 1, Herpes simplex 2, Varicella-zoster virus, Epstein-Barr virus, Respiratory syncytial virus, Chikungunya, Dengue, H. Pylori, and Mycoplasma pneumonia). As equivocal results in previous studies of single infections may be due to confounding by other pathogenic infections and the modifying effect of age, we investigated the association of a pathogenic load of multiple latent infections on measures of prevalent frailty and mortality in a study sample of young-old and old adults.

## RESULTS

**Figure 1:**
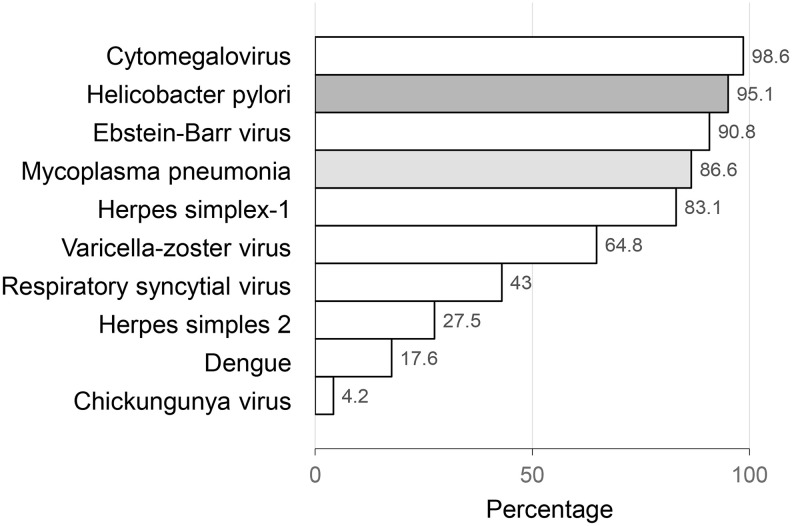
**Latent infections by pathogens.**

The mean age of the study participants was 72.7 years (range: 57-92). The mean PL count was 6.1 (SD=1.2), individually ranging from 3 to 9. The most common pathogens (CMV, H. Pylori, EBV, M. Pneumoniae, and HSV-1) were prevalent in 80 to 99% of the participants, while other pathogens were prevalent in less than 65% of the participants, with the least prevalent pathogen being Chikungunya virus (4.2%). Figure 1.

The mean CD-FI scores was 0.26 (SD=0.086, range: 0.09 to 0.49); 54.2% were pre-frail (1-2 components), and 14.1% were frail (3-5 components). Participants with high PL (7-9) were significantly older, and tended to show more smoking histories, to live alone, have greater number of chronic diseases, and higher CRP levels, although the differences were not statistically significant at p<0.05. Table 1. However, they showed significantly greater mean scores on the CD-FI (p=0.001 and p=0.002), compared to those with lower PL. A quarter (24.6%) of those with high PL were physically frail (PP-FI score of 3-5), significantly more than their low PL counterparts (6.2%, p=0.001). They were significantly more likely to be weak (57.4%) compared to the low PL counterparts (29.6%, p=0.001), and to be slow (23.0%) compared to their counterparts (8.6%), p=0.017. Otherwise, there were no significant differences for exhaustion, inactivity or shrinking.

**Table 1:** Demographic, clinical, physical, cognitive and mental functions, and survival status of study participants by pathogenic load categories.

	**Pathogenic load**		**Significance P**
	**3-6**	**7-9**	
Participants, N	81	61	
Male sex	60.5 (49)	57.4 (35)	0.71
Age, years	71.5 ± 8.0	74.5 ± 8.6	0.036
Less than 7 years of education	70.4 (57)	68.9 (42)	0.84
Current smoker	6.2 (5)	14.8 (9)	0.16
Daily alcohol drinker	3.7 (3)	4.9 (3)	0.72
Living alone	46.9 (38)	54.1 (33)	0.40
No. of medical illnesses	2.69 ± 1.59	3.03 ± 1.89	0.24
BMI, kg/m^2^	23.3 ± 4.1	23.2 ± 5.1	0.93
CRP, mg/L, median (IQR	0.287 (0.103 – 0.775)	0.502 (0.186 –0. 826)	0.099^†^
CD-FI Frailty Index	0.240 ± 0.074	0.286 ± 0.095	0.001
Robust (0)	40.7 (33)	19.7 (12)	
Pre-frail (1-2)	53.1 (43)	55.7 (34)	
Frail (3-5)	6.2 (5)	24.6 (15)	0.001
Weakness	29.6 (24)	57.4 (35)	0.001
Slowness	8.6 (7)	23.0 (14)	0.017
Exhaustion	11.1 (9)	23.0 (14)	0.058
Inactivity	33.3 (27)	36.1 (22)	0.735
Shrinking	13.9 (11)	16.4 (10)	0.685
Deaths	62.5 (50)	81.7 (49)	0.014

Figures shown are % (N) or Mean ± SD

P values from t-test of significance for continuous variables and chi-squared tests for categorical variables

^†^ Non-parametric significance test (Mann-Whitney U)

**Figure 2:**
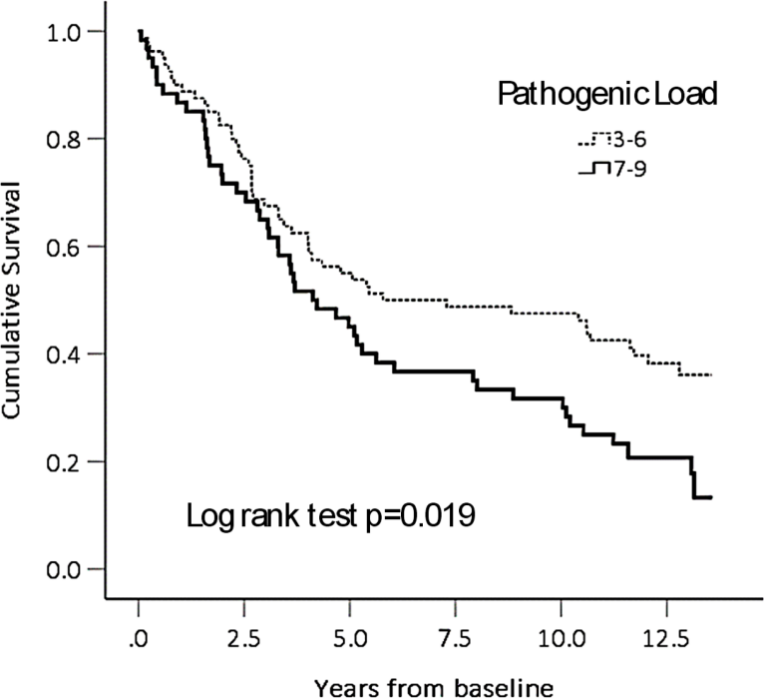
**Cumulative survival by pathogenic load category.**

The estimated effect sizes of association of high PL with these frailty measures are shown in Table 2. Controlling for the confounding influences of sex, age, education, smoking and alcohol history, these associations were attenuated but remained statistically significant. A high PL was associated with an estimated increase of 0.035 points in the CD-FI (Cohen’s D=0.035 / 0.086, or 0.41). A high PL was associated with 8.5 times odds of being physically frail (p=0.001), 2.8 times odds of being weak (p=0.010); and 3.4 times odds of being slow (p=0.024). The mortality hazard ratio associated with a high PL, controlling for age, sex, education, smoking and alcohol histories, was 1.53 (95% CI, 1.01-2.1). See Figure 2.

**Table 2:** Estimated effects of pathogenic load on frailty and mortality outcomes.

	**Unadjusted OR (95% CI)**				**Adjusted OR (95%CI)**		
	**PL=3-6**	**PL=7-9**	**P**		**PL=3-6**	**PL=7-9**	**P**
	**N=81**	**N=61**			**N=81**	**N=61**	
Frailty Index (0-1)	0 (reference)	0.046 (0.018, 0.074)	0.001		0 (Reference)	0.035 (0.007, 0.063)	0.015
Prefrail (vs Robust)	1 (reference)	2.17 (0.98, 4.84)	0.057		1 (reference)	1.86 (0.77, 4.49)	0.166
Frail (vs Robust)	1 (reference)	8.25 (2.46, 27.6)	0.001		1 (reference)	8.54 (2.32, 31.5)	0.001
Weakness	1 (reference)	3.08 (1.53, 6.20)	0.002		1 (Reference)	2.84 (1.28, 6.28)	0.010
Slowness	1 (reference)	3.15 (1.18, 8.37)	0.022		1 (Reference)	3.40 (1.17, 9.87)	0.024
Exhaustion	1 (reference)	2.43 (0.97, 6.08)	0.057		1 (Reference)	2.45 (0.91, 6.62)	0.076
Inactivity	1 (reference)	1.13 (0.56, 2.27)	0.735		1 (Reference)	1.19 (0.57, 2.46)	0.647
Shrinking	1 (reference)	1.21 (0.48, 3.07)	0.685		1 (Reference)	0.99 (0.35, 2.77)	0.985
Mortality hazard ratio	1 (reference)	1.60 (1.08, 2.38)	0.019		1 (Reference)	1.53 (1.01, 2.31)	0.046

PL: pathogenic load

Figures shown are estimates of group mean differences in Frailty Index, or odds ratios of physical frailty and its components, and mortality hazard ratio, and their (95% confidence intervals).

Adjusted for sex, age, education, smoking and alcohol history

In additional analyses, we explored the association of latent infection by specific pathogens with frailty. We found no significant associations for all except marginal associations for EBV and Chikungunya. In preliminary analyses of variance, EBV seropositivity was significantly associated with PP-FI (mean ± SD of 1.27 ± SD 1.16 versus 0.62 ± 0.77, p=0.012), but the association was not significant (p=0.095) when controlled for confounding variables. Chikungunya seropositivity was associated with CD-FI (mean ± SD, 0.329 ± 0.108 versus 0.257 ± 0.084, p=0.014, but the association was not significant (p=0.053) when controlled for confounding variables. We also compared participants with high anti-CMV IgG titers in the top tertile range versus those with low titers, and observed no difference in CD-FI (0.256±SD 0.083 vs 0.266 ±SD 0.092, p=0.546).

## DISCUSSION

To our knowledge, this is the first study that links a high pathogenic load of latent infections to an increased risk of frailty and mortality. Thus far, only a handful of studies have investigated a link with frailty for latent infection by a few individual pathogens of interest, namely CMV, EBV, VZV and HSV, and in single order [16–18, 26, 27, 29]. We found notably in this study population sample that virtually all but 2 participants were tested positive on CMV serology, and neither CMV seropositivity nor high antibody titers was associated with frailty. Hence for latent infection by pathogens like CMV, H. Pylori, EBV, M. Pneumoniae, and HSV-1 that are ubiquitous in most populations including ours, it will be exceptional for studies to detect a significant link with frailty. The same may apply for rare infections by pathogens such as Chikungunya in this population, unless the study has a very large sample size. In this study we took a different line of inquiry away from the traditional approach of establishing unique associations with frailty for specific pathogenic infection on its own. Instead we hypothesized that multiple latent infections by a variety of pathogens each with varying small effects may contribute collectively to a large effect on frailty and mortality when present in the same individual.

By the nature of latent infection, the potential reactivation of pathogens is kept in check by constant immune surveillance, and wherewith, the induced responses of virus-specific CD8+T cells can be detected long after the virus is controlled [37]. In HIV disease where a basal viral load is constantly present, or CMV infection which intermittently reactivates during the lifetime, constant or repeated viral replication and antigenic stimulation have been shown to cause an antigen-dependent clonal expansion of the memory T cells resembling immunosenescence. Most CMV-specific CD8+ T cells are of TEMRA phenotype with decreased CD28, CD27, CCR7, CD62L and increased CD57 and re-expressed CD45RA surface markers, and are largely pro-inflammatory producing a large amount of IL-6, TNFa, and IL-1b [38]. We have previously reported an association between physical frailty and markers of T cell senescence [32] and herewith we provide evidence for immunological history linking this previous observation. The clinically latent or asymptomatic viral disease under viral load control is thus characterized by a persistent systemic inflammation, which may thus contribute to age-associated diseases and the frailty state. Also, the maintenance of viral infection in the latent state carries a high energy cost, as the virus reactivation potency [39] and the expansion of CD8+ T cells at the expense of other T cell subpopulations both rely on the host’s mTOR pathway, thus depleting energy reserves, which also characterizes the frailty state.

The immune system response to chronic stress originating from latent infection represent an allostatic process to maintain homeostasis [3]. The allostatic overload from chronic antigenic stimulation from pathogenic sources is akin to the multisystem physiological dysregulation that researchers believe underlies ageing and frailty. Studies show that higher allostatic load scores based the count of multiple biomarkers of cardiovascular, endocrine, metabolic and immune function are associated with higher levels of the physical frailty syndrome [5]. Our finding of the link between pathogenic load and frailty and mortality in this study may thus be understood in the same context.

Our study does not exclude the possibility that certain specific viral agents such as CMV may be linked to frailty at least in some populations. Rather, it supports the allostatic load hypothesis that the increasing numbers of latent infection from different pathogens including viruses, bacterial and mycoplasma was associated with increased frailty and mortality risks. General and perhaps varied processes of immune surveillance responses to multiple pathogen-specific antigenic stimulations may cause gradual erosion of the immune system and other systems over the lifetime, and explain the development of age-related frailty. The caveat to this generalization is that while viruses such as CMV, HIV and HBV are likely to drive the accumulation of memory T cells towards senescence as described above, other viruses such as EBV and VZV as well as non-viral pathogens with their different life cycle and pathophysiology [40, 41] may have different mechanisms, which have not been as well studied.

There are some limitations in this study to note. Serological testing was performed according to manufacturer’s instructions and seropositivity was determined according to the antibodies titre cut-offs provided by the manufacturer. A lack of test standardization between laboratories and variations in cut-off levels may affect the sensitivity and specificity for a specific serological test. Variations in sensitivity and specificity across different serological tests may result in varying seropositivity leading to under-counting or over-counting of the pathogenic load. While serologic testing indicate previous infection was present at some time prior to 2003, it does not provide information on the duration of infection. Hence, it does not perfectly measure the degree of antigenic stimulation that the donor has been experiencing over a long period of time.

Additionally, there may have been additional infections during the period of follow up from 2003 to 2017, that were not captured by the serological data. Our study has the limitation of small sample size and case-control design, with the possibility of inherent prevalence bias and the odds ratio over-estimation of risks. We thus suggest that our results be viewed as preliminary findings and larger prospective cohort studies should be conducted.

## MATERIALS AND METHODS

### Study design and participants

The study involved community-dwelling older persons aged 55 and above who were participants in the first wave recruitment cohort (N=2804) in the Singapore Longitudinal Ageing Study (SLAS-1), who had baseline assessments and blood drawn in 2003 to 2004, and followed up for mortality outcome using the National Death Registry at the National Disease Registry Office using computerized matching search for date and causes of death. Previous publications have detailed the SLAS study design, population sampling and measurements [31]. The research was approved by the National University of Singapore Institutional Review Board, and informed consent was obtained from all participants.

In a sub-study of ageing immunology [32], we conducted a nested case-control study involving a total of 100 cases who died during the follow up period till 31 March 2017, and 44 surviving controls who were recruited contemporaneously during the same period of time as the case subjects. The controls at baseline had no reported history of recent hospitalization in the previous 6 months and non-elevated levels of C-reactive proteins (<10mg/L) due to significant inflammation from an infectious or non-infectious cause. (All control specimens had values below 4.3 mg/L in this study at the time of baseline assessment). We used stored blood samples collected at baseline to perform serological testing for all but two of the participants, hence the study sample involved a total of 142 participants.

At baseline, extensive structured questionnaire interviews, clinical, blood and performance testing were performed to collect demographic, medical history, physiological, cognitive, physical and functional measurements, that included age, sex, education, smoking and alcohol history, living arrangement, medical history (presence of twenty named and other medical disorders), number of chronic illnesses, nutritional status, body mass index, balance and gait (Performance Oriented Mobility Assessment (POMA) score, Mini Mental State Examination (MMSE) global cognition score, Geriatric Depression Scale (GDS-15) measure of depressive symptoms, 12-item Short Form Physical Component Score (SF12-PCS) and Mental Component Score (SF12-MCS) measures of health-related quality of life, functional status (basic and instrumental activity of daily living index).

*Cumulated Deficits Frailty Index (CD-FI)* counts the number of health deficits a person has and divides this by the total number of deficits evaluated to give a fractional score between 0 and 1. The CD-FI at baseline was derived using a total of 87 evaluable health deficit items in individual participants based on information on the presence of chronic diseases, physical, psychological, social and functional deficits, and abnormal blood test results, including items such as poor self-rated health, depressive symptoms, cognitive impairment, poor nutrition status, respiratory symptoms, FEV_1_/FVC<0.70, hearing and visual loss, overweight-obesity, hypertension, hypotension, lipid abnormalities, hypoalbuminaemia, anaemia, low estimated glomerular filtration rates, difficulty with eating, dressing, mobility, and other daily living activities, limitations in physical, social and occupational activities. (See list of evaluated deficits in Supplementary Tables 1 and 2)

*Physical frailty (PF) phenotype* at baseline was derived from assessments and criteria proposed by Fried and colleagues used in the Cardiovascular Health Study (CHS) (1), with operational modifications:

1. Shrinking was defined as weight loss of >=4 kg in the past 6 months or BMI of<18.5 kg/m2;
2. Muscle weakness was assessed as part of the Tinetti Performance Oriented Mobility Assessment (POMA) [33] by performance on rising from a chair sitting position with arms folded, using standards coring criteria. The summed score (range: 0-16) in the lowest gender- and BMI- adjusted quintile was used as cut-offs.
3. Slowness was determined with the subject walking 6 meters and returning quickly to the starting point, and defined by gait performance scores (range: 0 to 12) of less than 9. [33]
4. Exhaustion was measured by one question from SF-12 quality of life scale [34]:”Do you have lots of energy”
5. Low activity was assessed by questions on the number and frequencies of usual participation in 16 categories of activities using 3-point Likert scale for each activity, and low activity was defined as a total score below the lowest gender-adjusted quintile.

By assigning 1 point for the presence of each frailty component, a summed score (0-5) was derived to categorize subjects as frail (score=3-5), pre-frail (score=1 or 2) and robust (score=0 point). This modified measure of the physical frailty showed good agreement (weighted kappa 0.63) with a measure which used measures of knee extension strength and fast gait speed in a second SLAS cohort (SLAS-2) and previous studies have shown it to be equally and strongly predictive of adverse health outcomes including IADL-BADL dependency, hospitalization, poor quality of life, dementia and mortality [35, 36].

### Serological testing

Overnight fasting blood was tested serologically for IgG antibody levels via enzyme-linked immunosorbent assays (ELISA) to ten infections that are locally important or highly prevalent among Asian populations: Cytomegalovirus (Genesis Diagnostics, United Kingdom), Dengue (Diagnostic Automation, USA, California), Herpes simplex 1, Herpes simplex 2, Varicella-zoster virus, Epstein-Barr virus (VCA), Respiratory syncytial virus, Chikungunya, H. Pylori, and Mycoplasma pneumonia (Novatec Immundiagnostoca GmBH, Germany). The sensitivities and specificities of these tests range from 80% to 100%. The ELISAs were carried out according to manufacturer’s instructions. Plasma samples were incubated in wells of ready-coated plates coated with viral or bacterial antigens, binding antibodies in plasma that are specific for these antigens. After washing, the bound antibodies were incubated with enzyme conjugates and an enzymatic reaction resulted upon addition of a chromogen substrate. Reaction was terminated and the absorbance was measured immediately on the EnVision® 2104 multimode microplate reader (Perkin Elmer). Seropositivity was determined according to the antibodies titre cut-offs provided by the manufacturer. (Supplementary Tables 1, 2).

The number of serological tests that were positive for the 10 pathogens in the individual were added to give a cumulative score of the pathogenic load from multiple latent infections, which ranged from 3 to 9 in this study sample of participants. We used the approximate median count to categorize study participants accordingly to those with low pathogenic load count (3-6) and high pathogenic load count (7-9).

### Data analysis

Differences in demographic and clinical variables between the two pathogenic load groups were assessed using χ2 tests for categorical variables and analyses of variance (t-test) for continuous variables. Comparisons of the mean scores of the CD-FI and the estimated differences between the two pathogenic load groups were performed using analyses of variance in regression analyses that controlled for sex, age, education, smoking and alcohol history. Estimates of the associations between pathogenic load and the prevalence of the physical phenotype components were performed using logistic regression, controlling for the same confounding variables. Cox regression analysis was used to estimate the hazard ratio of association between pathogenic load and mortality, controlling for confounding variables. All analyses were performed using IBM SPSS statistical software version 25.

## Supplementary Material

Supplementary Tables
