# Supplementary material for: Pathogenic load and frailty in older adults: Singapore longitudinal ageing study
Source: Aging (Albany NY). 2020 Nov 6;12(21):22139–51. doi: 10.18632/aging.104076 (PMC7695406; doi:10.18632/aging.104076)
Supplement: Supplementary Tables [file aging-12-104076-s001..pdf]

SUPPLEMENTARY TABLES

Supplementary Table 1. List of 87 binary variables used to derive Frailty Index.

	Item
1	Live alone
2	No body able to help if you needed/wanted
3	Need help with finances
4	Single divorced widowed
5	Hearing problem
6	Low Visual function (VF14<100)
7	Depression GDS >=5
8	Cognitive impairment MMSE<23
9	Physically prefrail or frail (CHS criteria)
10	Wide waist circumference
11	Hypertension (Self-report, medication, high sitting BP
12	Dyslipidaemia (self-report, abnormal lipid panel, medication)
13	Diabetes (self-report, medication, elevated FBG
14	Orthostatic hypotension
15	History of stroke
16	History of myocardial infarct
17	History of atrial fibrillation
18	History of heart failure
19	History of cataracts/major eye problems
20	History of kidney failure on dialysis
21	History of asthma
22	History of chronic obstructive lung disease
23	History of arthritis
24	History of hip fracture
25	History of mental illness
26	History of any other problems
27	History of cancer
28	Hospitalized in past one year
29	Fall(s) in past 6 months
30	Treated for Injury in past one year
31	Anaemia (Hb<13 in male, <12 in female)
32	Low Albumin <40mg/dL
33	Chronic kidney disease (eGFR<60 ml/min
34	Low BMI (<18.5)
35	Overweight/Obese (BMI >27.5k/m2)
36	Illness changes the kind/amount of food eaten
37	Fewer than 2 meals eaten per day
38	Few fruits/vegetables/milk products eaten (less than 1/day)
39	3 or more drinks of alcohol daily
40	Eating difficulty due to tooth or oral problems
41	Not enough money to buy needed food
42	Eat alone most of the time
43	5 or more different prescribed/over the counter drugs a day
44	Unintended weight loss 10lbs/4kg last 6 months

45	Unintended weight gain10lbs/4kg last 6 months
46	Not physically able to shop, cook and/or feed myself
47	Eat few milk products (less than once a day)
48	Chronic airway obstruction (FEV1/FVC <0.70)
49	Cough on getting up in the morning
50	Cough during the day or night
51	Cough over 3 months in the year
52	Phlegm on getting up in the morning
53	Phlegm during the day or night
54	Phlegm over 3 months in the year
55	Cough with phlegm for 3 consecutive months in past year
56	Increased cough/phlegm for 3 weeks or more in past 3 years
57	Breathless on mild exertion
58	Need help with bathing
59	Need help with dressing
60	Need help transfer from bed to chair and back
61	Need help walking around the house
62	Need help eating
63	Need help with grooming
64	Need help with using toilet
65	Need help getting up and down stairs
66	Need help with shopping
67	Need help with housework
68	Need help using phone
69	Need help with meal preparations
70	Need help with taking medication
71	Feel everything I do is an effort
72	Poor general health
73	Limited in doing moderate activities during typical day
74	Limited in climbing stairs during typical day
75	Limited in accomplishing lesser than would like
76	Limited in work/other activities
77	Emotional problems - accomplished lesser
78	Emotional problems interfere with work or other activities
79	Pain interfere with normal work
80	Felt calm & peaceful none of the time
81	Have lots of energy none of the time
82	Felt downhearted and low past 4 wks
83	Physical/emotional problems interfere with social activities
84	Life is fairly or very boring
85	Life is fairly or very sad
86	Life is fairly or very hard
87	Feel lonely

Supplementary Table 2. Serological tests.

ELISA kit	Company	Units	Range	Negative	Grey zone	Positive
Cytomegalovirus	Genesis Diagnostics	IU/ml	0-30 IU/ml	OD < 3 IU/ml standard OD	NA	OD >/= 3 IU/ml standard OD
Dengue	Diagnostic Automation	NA	NA	0 - 0.15 OD units	NA	Weakly positive: 0.16 - 1.0 OD units<br>Strongly positive: >1.0 OD units
Herpes simplex 1	Novatec	NTU	NA	OD is lower than 10% below the cut-off	OD of 10% above or below the cut-off	OD is higher than 10% over the cut-off
Herpes simplex 2	Novatec	NTU	NA			
Varicella-zoster virus	Novatec	NTU	NA			
Epstein-Barr virus (VCA)	Novatec	NTU	NA			
Respiratory syncytial virus	Novatec	NTU	NA			
Chikungunya	Novatec	NTU	NA			
Mycoplasma pneumonia	Novatec	NTU	NA			
H. Pylori	Novatec	NTU	0 - 150 NTU/ml	< 15 NTU/ml	15 - 20 NTU/ml	> 20 NTU/ml

*NTU = Novatec Units
